# Practical macromolecular cryocrystallography

**DOI:** 10.1107/S2053230X15008304

**Published:** 2015-05-27

**Authors:** J. W. Pflugrath

**Affiliations:** a Rigaku Americas Corp., 9009 New Trails Drive, The Woodlands, TX 77381, USA

**Keywords:** cryocrystallography, cryoprotectant, flash-cooling, crystal mounting, annealing, automounter, high pressure

## Abstract

Cryocrystallography is an indispensable technique that is routinely used for single-crystal X-ray diffraction data collection at temperatures near 100 K, where radiation damage is mitigated. Modern procedures and tools to cryoprotect and rapidly cool macromolecular crystals with a significant solvent fraction to below the glass-transition phase of water are reviewed. Reagents and methods to help prevent the stresses that damage crystals when flash-cooling are described. A method of using isopentane to assess whether cryogenic temperatures have been preserved when dismounting screened crystals is also presented.

## Abstract 2

Current methods, reagents and experimental hardware for successfully and reproducibly flash-cooling macromolecular crystals to cryogenic temperatures for X-ray diffraction data collection are reviewed.

## 1. Introduction   

Macromolecular crystal structure determination has benefited enormously from the reduction of damaging radiation effects on crystals that is obtained by maintaining the crystals at cryogenic temperatures of around 100 K during diffraction data-collection experiments. Without flash-cooling and other cryocrystallographic methods, the crystal structure determination of proteins, oligonucleotides, their complexes and other large macromolecules would be much more difficult and time-consuming than is currently practiced. In 2014, the vast majority of diffraction data sets for crystal structure models deposited in the PDB were reported to have been collected at cryogenic temperatures, which is consistent with earlier reports (Berman *et al.*, 2000 ▶; Garman & Owen, 2006 ▶). Nowadays, the choice for X-ray data-collection temperature is below 120 K, which was not the case just a few decades ago, when room-temperature data collection predominated.

The early protein crystallographers observed radiation damage during their room-temperature diffraction experiments (Blake & Phillips, 1962 ▶). Cooling of lactate dehydro­genase crystals during the diffraction experiment was explored by Haas & Rossmann (1970 ▶), who suggested

the freezing of the mother liquor in the crystal’s interstices would retard the transport of free radicals through these regions.

They also went on to note that

freezing of protein crystals normally leads to crystal cracking owing to the differences in volume in the included water and ice (Low, Chen, Berger, Singman & Pletcher, 1966 ▶) and, thus, to a gross deterioration of the diffraction pattern.

In their study, they used both glycerol and sucrose as cryoprotectants. They also wrote that

[i]t was found essential to freeze the protein crystals rapidly and isotropically.

The result was a tenfold reduction in the rate of radiation damage and the avoidance of background scatter from the typical capillary-mount method. Thus, this seminal article on cryocrystallography predicted all the elements of experiments performed 45 years into the future.

Along the way to the future, Petsko (1975 ▶) showed how cryoprotectant solutions could be exchanged with mother liquor ‘for maintaining the integrity of protein crystals at sub-zero temperatures’. Hope (1988 ▶) introduced a kind of micromount spatula made from thin glass shards and used it for ribosome cryocrystallography (Hope *et al.*, 1989 ▶). Teng (1990 ▶) described that loops made from fine-gauge wire for mounting thin plate-like crystals helped to prevent an increase in the mosaicity of such crystals. While both home-made and commercial low-temperature open-flow cryosystems that used the boil-off gas from a Dewar of liquid nitrogen were available in the 1970s and even earlier, not until the late 1980s to the 1990s did they have a standard presence in the macromolecular crystallography laboratory with the introduction of a reliable, low-use nitrogen cryosystem (Cosier & Glazer, 1986 ▶). All of these development efforts coincided with the commissioning of modern synchrotron sources with their high-brilliance X-ray beamlines and their ability to swiftly damage crystals with ionizing radiation.

Several previous reviews of cryocrystallographic methods are available, and readers are encouraged to consult them since each one contains helpful tips from a unique perspective, both historical and experimental (Rodgers, 1997 ▶; Garman & Schneider, 1997 ▶; Parkin & Hope, 1998 ▶; Garman, 1999 ▶; Pflugrath, 2004 ▶; Garman & Owen, 2006 ▶, 2007 ▶; Hope & Parkin, 2012 ▶; Rodgers, 2012 ▶). In addition, cryotechniques were used even earlier in electron microscopy (Steinbrecht & Zierold, 1987 ▶). For this review it is unfair to cite only a few sources, but it is also difficult to cite them all.

Since cryoprotecting and mounting fragile crystals is not yet reliably (not to mention cost-effectively) automated (Deller & Rupp, 2014 ▶), crystallographers need to be adept and confident with the techniques of sample preparation for cryocrystallography in order to be efficient and successful. This article reviews the current state of cryocrystallographic methods for diffraction data collection.

### 1.1. The need for cryocrystallography   

When crystals are exposed to ionizing radiation during the X-ray diffraction data-collection experiment they are damaged, as evident from the changes in the diffraction patterns that are observed over time. A thorough treatment of the causes and effects of radiation damage are beyond the scope of this article, so the reader is directed to other articles on this subject such as Nave & Garman (2005 ▶), Murray *et al.* (2005 ▶), Holton (2009 ▶), Garman (2010 ▶), Warkentin, Hopkins *et al.* (2013 ▶) and others. Nevertheless, a brief summary is presented. Some of the energetic X-ray photons impinging on the crystal sample will be absorbed and interact with the atoms therein and create photoelectrons, free radicals and charged species. These chemical changes will in turn create other secondary changes in a cascading effect. For instance, water molecules, hydroxyl ions, protons and free radicals can interact with protein atoms and reduce side chains, especially those containing sulfur. Metals can be reduced. Carboxylates can be decomposed by the expulsion of a CO_2_ molecule. Halides can interact with the reactive species and no longer interact in the same way with the surface of a protein as before. All of these changes can manifest themselves in changes in the positions of the atoms of the macromolecules, solvent and ligands found in the crystal. Warkentin, Hopkins *et al.* (2013 ▶) have noted that chemical changes cause local conformational relaxations in flexible loops and side chains, which in turn cause structural relaxations on a variety of length scales which are a dominant contribution to radiation damage at room temperature.

Thus, the structure of the crystal changes with X-ray dose (energy absorbed per unit of mass). The structure is not the same before and after X-ray exposure, and thus the X-ray diffraction pattern changes with dose and radiation damage.

Protein crystals can absorb a limited X-ray dose before they become unusably damaged. The tolerable dose will depend on the specific experiment and the desired resolution limit of the usable diffraction data. Henderson (1990 ▶) predicted that a dose of 20 MGy would destroy the diffraction from protein crystals. Owen *et al.* (2006 ▶) experimentally determined larger tolerable limits for crystals maintained at 100 K and recommended an absorbed dose limit of 30 MGy. Howells *et al.* (2009 ▶) compiled estimates of the maximum tolerable dose for X-ray diffraction microscopy and crystallography of protein crystals. For the resolution range 0.1–10 nm they suggested a linear relationship in their equation (5) of a maximum tolerable dose limit of 10 MGy per desired resolution in angstroms. That is, 30 MGy for desired 3 Å resolution diffraction data, 20 MGy for 2 Å and 10 MGy for 1 Å.

The advantage of diffraction data collection at cryogenic temperatures is not only that the damage directly caused by radiation is prevented, as this still occurs. Other effects are also significantly diminished simply because the relatively large reactive species cannot freely move as the entire sample is encased in a vitreous glass-like phase where no major motions are possible. Furthermore, all of the atoms are trapped by their neighboring atoms, which also cannot move. This is clearly in contrast to data collection at room temperature, where the water molecules and reactive species in a crystal lattice are free to diffuse around in the internal solvent channels, within the protein itself and on the surface of the crystal. One can appreciate that electrons are free to quantum-mechanically tunnel and diffuse at cryogenic temperatures and still cause secondary damage effects. Indeed, Owen *et al.* (2012 ▶) report that the larger free radical OH* is still mobile above 110 K.

**Figure 1:**
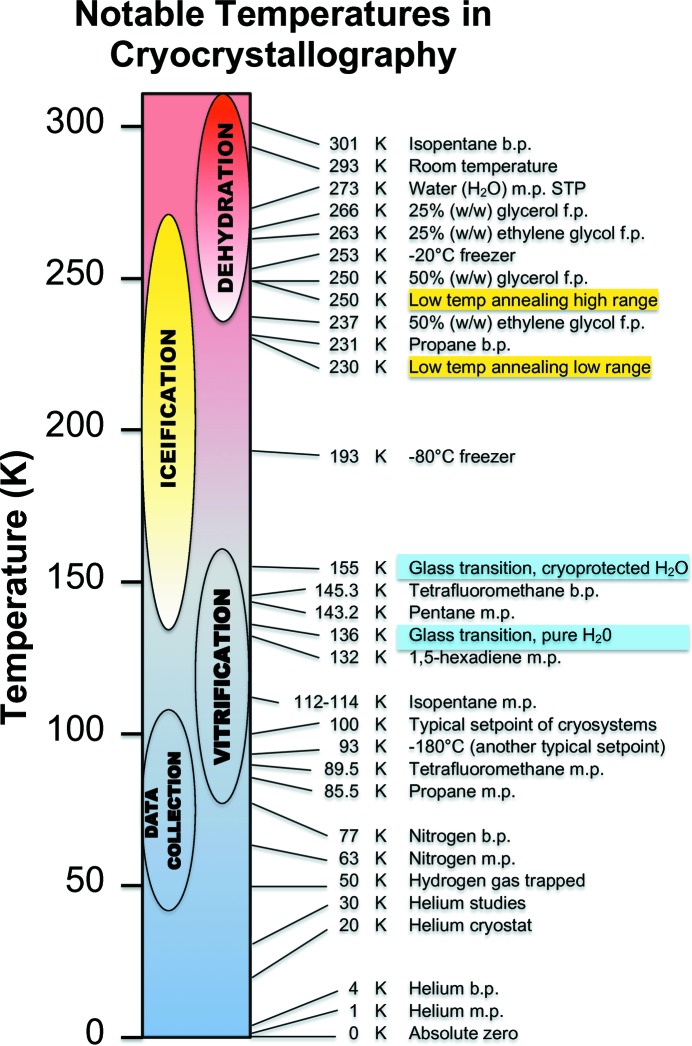
Notable temperatures in cryocrystallography. A vertical temperature scale is colored with four major regions marked: (i) dehydration, where crystals are damaged by drying, (ii) iceification, where crystals are damaged by ice formation when freezing, (iii) vitrification, where ice does not form when suitable cryoprotectants and adequate speed in flash-cooling are used, and (iv) data collection, where diffraction experiments are typically performed. Abbreviations used: m.p., melting point; f.p., freezing point; b.p., boiling point; STP, standard temperature and pressure. Glycerol solution temperature points are from Lane (1925 ▶). Ethylene glycol solution temperature points are from Cordray *et al.* (1996 ▶). Other temperature points are referred to in the text.

Flash-cooling a crystal to a temperature below the glass-transition phase (∼136–155 K; Weik, Kryger *et al.*, 2001 ▶; Weik, Ravelli *et al.*, 2001 ▶; Chinte *et al.*, 2005 ▶; see Fig. 1 ▶) is the method used to trap the molecules and atoms in a vitreous glass phase. Methods by which this is accomplished in a laboratory setting are explored below. However, it must be emphasized that this state in no way prevents radiation damage from occurring. It only mitigates some of the observable effects of the damage. Namely, if atoms and molecular subspecies created by ionizing radiation do not move away from their initial positions by tenths of an angstrom, then the apparent order in the crystal lattice is maintained at a resolution unaffected by the small positional changes. However, the order at high resolution no longer exists if parts of the molecules in the crystal have changed in position by too much. Thus, the loss of intensities in higher order Bragg diffraction spots is evidence of this loss of order caused by the changes in the positions of atom centers. In other words, loss of high-resolution diffraction is one outcome of radiation damage.

Further evidence that radiation damage still occurs at cryogenic temperatures can be seen when a crystal that has been exposed to X-rays at low temperature is warmed to a point where gross motion and diffusion of water and other small molecules are allowed. When the molecular species are no longer trapped in a vitreous phase and can move, it is not uncommon to observe that exposed crystals crack, gas bubbles form and the gases created even expand and ‘blow up’ the crystal (Teng & Moffat, 2000 ▶; Meents *et al.*, 2010 ▶).

In addition to the benefits obtained from reducing the observable effects of radiation damage, diffraction data collection at cryogenic temperatures has a host of other important advantages over room-temperature collection that should not be ignored.

(i) No mounting in capillaries, so often easier to mount.
(ii) No extraneous movement of the crystal if the loop is firmly mounted.
(iii) Potentially less extraneous mass in the X-ray beam, so X-ray backgrounds are reduced, which improves the signal-to-noise ratio of weak reflections.
(iv) Fewer crystals needed for a complete and multiple data set.
(v) One can mount, store and transport crystals when they are ready and there is no longer a need to worry about degradation occurring over time.

All of these advantages do not come without some accompanying disadvantages. One will need to become skilled at flash-cooling samples to prevent failures attributed to the disadvantages.

(i) Safety concerns.
(ii) Cost and storage of cryogenic liquids such as nitrogen, tetrafluoromethane and propane.
(iii) Cost of equipment: Dewars, cryosystems, tongs, vials and caps.
(iv) Cost of training and practice.
(v) Ice, on the crystal surface or internally.
(vi) Cryoprotectants are needed.
(vii) Increase in mosaicity, which is deadly for large unit cells.
(viii) Lack of isomorphism among multiple crystals or even over different volumes within a single crystal.

Since the advantages of cryocrystallography are well established, both the costs and the time devoted to mastering the techniques are well accepted nowadays. Thus, little additional justification is needed to obtain the funding to buy the equipment and paraphernalia needed to implement safe and consistently good techniques for cryocooling samples before data collection.

### 1.2. Safety   

Next, a word about safety. Human eyes, fingers and other body parts are not compatible with cryogenic temperatures, and should be protected from exposure to the safety hazards associated with cryocrystallography. Personal protective equipment such as safety glasses, face shields, gloves, laboratory coats and closed-toed shoes should be used at all times. Pressurized tanks of liquid nitrogen have their own safety concerns and should be treated with respect. One should be aware that the pressure-relief valves can open and release a cold gas jet near eye level, so when filling Dewars wear eye protection and work on the opposite side of the relief valve. Acceptable liquid-nitrogen transfer hoses should be steel double-jacketed and not be made of rubber. For filling open Dewars, the hose should have an attached liquid-nitrogen (LN_2_) phase separator to prevent the spraying of liquid nitrogen and cold gas. Dewars should be filled at floor level from these large tanks. Small desktop Dewars can be filled by pouring liquid nitrogen from smaller Dewars. Extra care must be taken with glass Dewars so that they do not break. One can first introduce a small amount of liquid nitrogen into them and swirl to pre-chill the inner surface evenly. Tall 2 litre Dewars for cryovial canes should be kept on the floor and not on the laboratory bench since they can so easily be knocked over. One recommendation is to put small Dewars in a Styrofoam bucket or shipping container as an added safety measure, since then they can be filled to the brim while overflowing liquid nitrogen stays in the secondary container. Another recommendation is to keep all Dewars containing liquid nitrogen covered as much as possible. Loose covers that extend down the outside of the lip of the Dewar are best since the outgassing of nitrogen keeps water vapor separated from the liquid nitrogen and reduces ice contamination. Tight covers are proscribed since they turn a Dewar into a potential bomb and they tend to freeze on the Dewar. Covers that do not extend down the side of the Dewar far enough build up ice at the Dewar lip, which always falls into the liquid nitrogen in the Dewar and eventually ends up on samples. Examples of this are shown in the videos that can be found at https://www.youtube.com/watch?v=1y89sfsuQBw and https://www.youtube.com/watch?v=45Qc3jOPaKY. Make every effort to prevent any tipping, tilting and sloshing of filled Dewars during transport from any filling location to the flash-cooling station.

Manual dexterity is not needed when filling Dewars, so that loose-fitting cryogenic gloves that can be thrown off are adequate to protect hands. When manipulating crystals in growth trays at the microscope during the actual flash-cooling, a double layer of a thin cotton or fabric glove for thermal protection covered by a laboratory nitrile glove for splash protection is typically used. Never use cotton or fabric gloves on their own. Human skin should not come in contact with any cryogenic liquid or even a piece of equipment at cold temperature. Safety eyewear is important since splashing, bubbling, burping and bumping of liquid nitrogen occurs. No one enjoys frozen eyeballs and clouding of the lenses of their eyes. Avoid articles of clothing that can trap spilled liquid nitrogen on skin as well.

Now that many of the safety issues have been addressed, one can turn to the issues of ice formation, which is the major cause of all the disadvantages of cryocrystallography. If crystals could be readily flash-cooled under all circumstances without any possibility of ice formation, then cryocrystallography would be even easier and trouble-free. Since this is not the current state of affairs, some expounding on the problem is warranted.

### 1.3. Theoretical background   

It has already been mentioned that if the atoms in the sample are kept from moving from their positions by being trapped in a vitreous phase, then the effects of radiation damage are minimized. In theory any dense solid phase should have the same function, so what is wrong with a solid ice phase? Water ice has lower density than normal water, so that if ice forms in the channels filled with solvent, the expansion changes the crystal lattice constants, disturbs the crystalline order, increases the effective crystal mosaicity in various ways and leads to reduced resolution of the diffracted Bragg reflections. Ice on the surface of the crystal has similar effects, and can also change the empirical absorption corrections applied to the reflections during data processing. Ice can also interfere with visualizing the crystal on the diffractometer, so that positioning the best part of the crystal in the X-ray beam is problematic. Furthermore, crystals of ice diffract and display a characteristic powder diffraction pattern of rings, which increases the X-ray background near these ice rings and may lead to poor estimates for affected Bragg reflection intensities. In summary, ice is just plain detrimental all round and introduces nonrandom and avoidable errors in the collected and processed diffraction spot intensities.

The process of flash-cooling or quenching crystals to cryogenic temperatures requires that the sample temperature drops quickly from above the freezing point of ice, passes through the glass-transition phase before appreciable ice can form, and the sample vitrifies (see Fig. 1 ▶). This is the basic guiding physical principle for the entire technique. Any methods that decrease the time for cooling to below the glass-transition temperature, which can be accomplished by increasing the rate of heat removed from the sample, by decreasing the total thermal mass of the sample and/or by lowering the rate of ice nucleation and growth *via* the addition of chemical cryoprotectants, are likely to be beneficial.

Cryoprotectants reduce the cooling rates required to achieve solvent vitrification mainly by suppression of ice nucleation. A much less important effect of cryoprotectants is to suppress the freezing point and increase the glass-transition temperature *T*_g_ (Weik, Kryger *et al.*, 2001 ▶; Garman & Owen, 2006 ▶). Warkentin, Sethna *et al.* (2013 ▶) discuss the minimum cooling rates required to avoid ice formation from a physical and theoretical viewpoint.

The rate of cooling can be increased by having a higher density of cryogen contacting the sample during the flash-cooling process. Liquid nitrogen is more dense than gaseous nitrogen. As a sample is plunged into liquid nitrogen, the heat from the sample boils the nitrogen and a microlayer of gas can develop around the sample (Gakhar & Wiencek, 2005 ▶). This gas layer can become substantial as the sample size and thermal mass increases. However, this gas layer created in LN_2_ is negligible compared with flash-cooling in a 100 K stream of cryogenic gas which is much less dense than liquid nitrogen. Cryogenic liquids with higher heat capacity and higher boiling points, such as propane and tetrafluoromethane, do not boil when used properly for flash-cooling samples, thus they may offer higher cooling rates (Teng & Moffat, 1998 ▶; Walker *et al.*, 1998 ▶; Kriminski *et al.*, 2003 ▶; Pflugrath, 2004 ▶; Edayathumangalam & Luger, 2005 ▶; Rodgers, 2012 ▶).

The larger the sample size, the more mass needs to be cooled. Studies have shown that smaller samples result in better results and lower cryoprotectant concentrations may be used (Warkentin *et al.*, 2006 ▶, 2008 ▶; Berejnov *et al.*, 2006 ▶). While crystallographers usually do not have the luxury of choosing the size of the crystals, they do have some control over the amount of extraneous mass included with their crystal when they quench to cryogenic temperatures. In particular, loop size and the amounts of carried-over solutions such as the mother liquor, cryoprotectant and oil should be minimized.

As a plunge occurs, one expects the crystal-enclosing solution to supercool before any ice nucleation occurs. This process can be defeated if extraneous ice nuclei are introduced during the flash-cooling time. For example, if microcrystals or nanocrystals of ice are already present in the liquid nitrogen or in the gas layers above it, then these ice crystals can seed the ice nucleation earlier than expected. This highlights the importance of keeping the Dewar contents free of ice (see below).

Knowledge of the physical principles can really help in explaining why flash-cooling is so variable among crystallo­graphers even if the crystals and cryoprotectant solutions are the same. Do they keep ice out of their Dewars? Do they remove excess solution from their crystals before the plunge? Do they remove the cold gas layer above the level of liquid nitrogen in their Dewars (Warkentin *et al.*, 2006 ▶), so that the sample avoids time spent at a temperature between the freezing point of water and the glass-transition temperature? (Fig. 1 ▶, iceification). Are they slow or quick?

The rate of cooling must be very high for all parts of the crystal so that there is no time for ice to nucleate and for ice crystals to appear. One tactic is to introduce solvents and compounds that lower the freezing point of the solvent. They disrupt the nucleation and formation of ice. These are called cryoprotectants and they will be discussed in detail shortly.

## 2. Hardware and devices   

**Figure 2:**
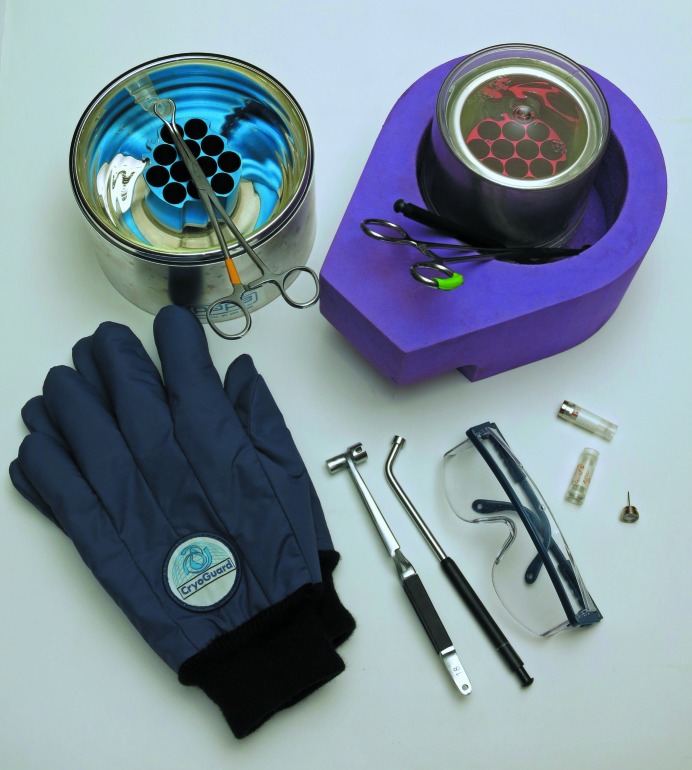
Accessories for cryocrystallography. Clockwise from lower left: gloves; shallow Dewar with automounter puck and clamping tongs holding a cryovial; purple foam Dewar holding a small glass Dewar with clear plastic cover; cryovials, cryomounting pin; safety glasses; curved magnetic wand; cryotongs.

A laboratory equipped for proper procedures of cryocrystallography will ensure the highest success rates in flash-cooling crystals. Besides the previously discussed safety considerations of personal protective equipment such as eyewear, face shields, gloves and double-jacketed steel transfer hoses equipped with phase separators, a laboratory will need a collection of Dewars, tongs, magnetic wands, racks and canes, and Dewar covers, as well as a standardized system of cryoloops, cryomounts, pins, bases, cryovials, pucks for sample changers and so on. Typical items are shown in Figs. 2 ▶, 3 ▶ and 4 ▶. Fortunately, there are many suppliers of pre-fabricated essentials to choose from, so that one is not forced to build one’s own system completely from scratch. A stereozoom microscope with a good working distance between the objective and sample trays is a prerequisite to view crystals.

A cryosystem, usually installed on a home-laboratory X-ray diffractometer with a viewing microscope, is very helpful to immediately test the results of flash-cooling of both potential cryoprotectant solutions and the crystals themselves (Fig. 5 ▶). Without such a cryosystem one would have to rely on shipping the flash-cooled samples in a dry-shipper Dewar to a synchrotron beamline in order to evaluate the results. A typical cryosystem blows cold nitrogen gas across the position of the crystal through a special nozzle which has a coaxial outer room-temperature flow of dry air or nitrogen that shields the inner cryogenic gas flow from the environmental water in the ambient atmosphere. The angle of the nozzle and associated coaxial gas streams are adjusted so that the inner cold gas stream contacts the crystal first before any turbulence causes mixing of the gases and any possible contamination of the crystal with ice or any temperature changes. The flow rates of the inner cold stream and the outer dry warm stream are also important to keep the sample free from gathering ice. Two typical nozzle orientations are used. (i) The nozzle may be oblique to the axis of the sample mount so that the gases blow past the goniometer head and goniometer, which keeps these pieces of experimental hardware from becoming cold and condensing and/or freezing water vapor on them, as shown in Fig. 5 ▶. (ii) The nozzle may be coaxial with the sample mount, but the goniometer head may need to be heated in order to reduce ice buildup. An advantage of the coaxial orientation is that the sample mount (loop stalk) will not vibrate differently as the crystal is rotated during the diffraction experiment owing to the relative change of the gas flow with respect to the loop and crystal. Another advantage is that the sample-mount pin base will remain very cold, which is helpful when dismounting samples. For all orientations, care should be taken so that the goniometer does not change temperature during the data collection, since if a portion moves into the downstream cold gas the ensuing temperature change will contract the metals and propagate a movement of the crystal away from the intersection of the X-ray beam and goniometer rotation axis. Also, the cryonozzle should not cast a shadow on the detector during data collection. Of course the nozzle must remain well aligned with the crystal position. There are alignment devices to help with this (Mitchell & Garman, 1994 ▶). Finally, the nozzle should be prevented from colliding with the detector and crystal goniometers during data collection. More information about nozzle positioning can be found in the work of Alkire *et al.* (2008 ▶, 2013 ▶).

If ice does form on the apparatus or on the crystal during data collection, then the cause should be found and remedied, although external ice may be removed as described later. Generally, ice on apparatus has an obvious solution, while ice on the crystal can be more problematic. Potential issues to check are (i) the flow rates of the inner and outer gas streams, which should be neither too high nor too low, (ii) the functioning of the outer concentric gas dryer, (iii) turbulence caused by a beamstop, collimator, microscope or sample mount in a gas stream, (iv) the distance of the nozzle from the sample (5–8 mm is typical), (v) air drafts (from air conditioning or heating) in the room, (vi) blockage of the gas flows from kinked tubing or even momentary blockage from pooling of liquid nitrogen in the gas path and (vii) relatively high ambient humidity. The nozzle position needs to allow the easy mounting of samples either by hand or *via* an automounter.

Fortunately, once a cryosystem has been set up properly, it should work flawlessly with only minimal maintenance such as filter and seal replacements and checking vacuum integrity, which is beyond the scope of this review.

**Figure 4:**
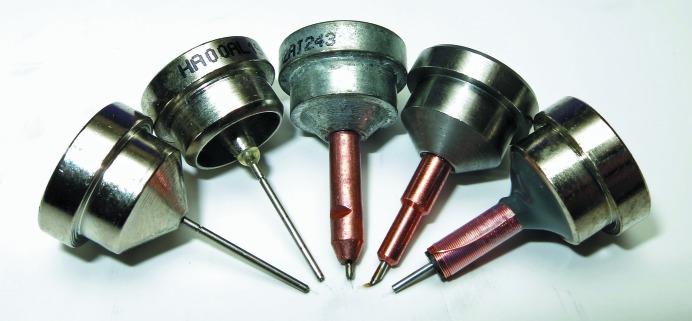
Close-up of five cryomounts. Left to right: Hampton Research ALS-style with steel pin; SPINE with steel pin; Hampton Research CryoCap copper with ledge; MiTeGen copper RT; Rigaku RFID mounting pin.

**Figure 5:**
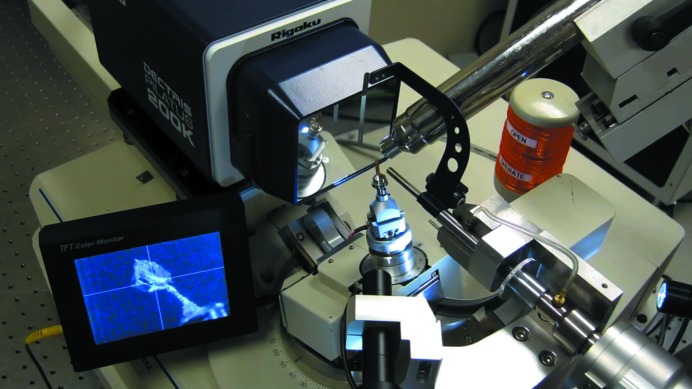
A typical X-ray diffractometer setup with a cryosystem. At the center is a crystal mounted on a pin magnetically held on a goniometer head. Clockwise from lower right corner: X-ray source collimator pointing towards the crystal with X-ray beamstop; black microscope; microscope display of the crystal in the cross-hairs mounted in a loop; X-ray detector; cryosystem nozzle pointing at the crystal; orange X-ray shutter indicator lamp. Note that the cryonozzle is pointing obliquely at the sample position so that the cryogenic gas impinges neither on the goniometer head nor on the X-ray detector, nor does it obscure the view of the crystal through the microscope.

Actual diffraction data collection rotates the crystal by means of a diffractometer that keeps the crystal in the X-ray beam and centered in the cryogenic gas flow. For this purpose, a traditional goniometer head is mounted on the diffracto­meter. The head has a magnet attached, onto which a steel or nickel metal base can be held by a magnetic force. The base has a smaller diameter steel or copper pin inserted into the base and into this pin is inserted a relatively X-ray transparent mounting loop or micromount. This small loop + pin + base combination is often simply called a base, a top hat, a cryocap, a cryomount or a mounting pin. Five commercially available cryomounts are shown in Fig. 4 ▶, although there are many other styles. The base is sized to fit partially inside a cryovial, so that when the loop end is inserted into the cryovial the base forms a cap and seals the cryovial. Cryovials are available with a magnet ring which holds the cap lightly in place during transport and mounting operations.

Not surprisingly, there are a variety of base + pin + loop configurations with features important for both manual and automated sample changers or robotic crystal mounters along with their associated sample holders or pucks. This is an active area of engineering design and improvement. The metal bases should not rust or oxidize. They should be labeled clearly with a unique identifier or barcode. They should bind tightly to the goniometer head magnet without tilting or slipping. They should not jam in the grip of a robot or its puck. They should conduct heat from the goniometer head magnet to prevent ice from building up on the pins during data collection. Bases with copper pins (diameter 3 mm) are better for data collections of several hours or days because they conduct heat better, while pins made of steel (diameter 1 mm) are better if the sample will be robotically dismounted and returned to a Dewar. This last point is important when screening crystals, a procedure which mounts and dismounts a crystal at least once before re-mounting for actual data collection. During a dismount a warm base and pin will increase the temperature of the crystal inside the end effector or gripper of a robot to even perhaps above the glass-transition temperature; thus, a base and pin with less mass will keep the transfer-environment temperature from rising too much and going above a critical point. Several sources of pre-made bases, pins and loop are available, including Hampton Research (Laguna Nigel, California, USA), MiTeGen (Ithaca, New York, USA), Molecular Dimensions (Newmarket, Suffolk, England) and others.

There are even more variations in the loops than in the bases and pins. Historically, loops were made of thin-gauge copper wire, Ethicon sutures, mohair fibers from a sweater and even the fine hair of a human baby. Except for the copper wire, the characteristic features of these were their low X-ray background and lack of fiber-diffraction features. Nowadays, loops are still made from nylon threads (usually 20 µm in diameter), but also polyimide films or resins (MiTeGen, Molecular Dimensions), which can be microfabricated into loops, meshes, nibs and other shapes. These microfabricated mounts provide a high level of consistency, making sample preparation easier. Of particular importance is that these mounting loops are mounted securely in the pins and do not vibrate owing to the physical forces from the cryosystem gas flow (see Flot *et al.*, 2006 ▶; Alkire *et al.*, 2013 ▶). Vibration and weathervaning have been shown to lead to systematic problems during diffraction data collection that usually are not discovered until the data-analysis steps.

As already mentioned, mounted samples on cryopins can be stored in cryovials and pucks for automated sample changers. Cryovials fit into labeled canes enclosed with plastic sleeves and then into canisters into Dewars. Several systems for holding robot pucks in racks that fit into Dewars are also available (Pflugrath, 2004 ▶; Owen *et al.*, 2004 ▶).

A variety of hand tools are also required to hold cryobases, to pick up microscopic crystals with loops or microdevices and to flash-cool them by plunging into cryogenic liquids (Figs. 2 ▶ and 3 ▶). A laboratory will need several sets of locking clamps for cryovials, magnetic wands for holding bases and pins, and cryotongs for transferring bases between Dewars, pucks and goniometer heads. Cryotongs are ingenious tongs that encapsulate a cryopin within a pre-chilled thick cylinder of metal (two halves of the cylinder separate to reveal a hollowed-out volume that precisely fits the base + pin + loop) and prevent contact with ambient temperature and air (Parkin & Hope, 1998 ▶). They allow samples under liquid nitrogen to be gripped safely, moved to the magnet on a goniometer head, released and exposed to the cryogenic gas of the cryosystem all without warming above the glass-transition temperature. A video of this procedure can be found at https://www.youtube.com/watch?v=1y89sfsuQBw. Such tongs form the basis of most of the automated sample changers in use today. These hand tools are available from the same vendors mentioned previously that supply cryobases and loops.

An assortment of Dewars complete the cryocrystallographer’s kit (Fig. 2 ▶). They run the gamut from small desktop Dewars to use when flash-cooling at a microscope, Dewars specifically designed to hold pucks when flash-cooling, taller 2 litre Dewars to hold canes of cryovials, 4 litre Dewars to fill from large LN_2_ tanks and to then fill the smaller desktop Dewars, large storage Dewars for holding canes and pucks, dry-shipper Dewars for sending samples to synchrotron beamlines, purpose-built Dewars for sample changers and so on (Pflugrath, 2004 ▶). Dewars can be made from glass, stainless steel or foam. The latter two are safer to use and may last longer in a laboratory. A variety of racks that fit into Dewars are also available.

A considerable number of words have already been expended on describing the deleterious effects of ice, but keeping Dewars and the liquid nitrogen they contain free from ice deserves some more words. Much of the ice that ends up in Dewars comes from water vapor in the immediate atmosphere; therefore, one needs to create a good barrier between ambient air and the inside of the Dewar. This barrier can be a physical cover, a layer of dry gas or both. A piece of foil, a flat piece of foam or a piece of cork all make terrible covers. A better cover sits tight on the top of the Dewar lip, but fits loosely along the outer walls and extends down over the edge a few centimetres (one is shown in Fig. 2 ▶).

With the experimental apparatus and tools necessary for a successful flash-cooling session, one should take care that the cryosystem is properly aligned and maintains the proper temperature at the sample position to ensure that it actually is below the lower range of the glass-transition phase and preferably below 100 K to help prevent OH radicals from diffusing. A melting-point determination of isopentane can be used first to calibrate the cryosystem temperature. WARNING: isopentane is extremely volatile and highly flammable at room temperature, so handle it with extreme care and use a very small volume transferred first in a fume hood. This is easily performed by mounting about a microlitre of liquid isopentane on a cryoloop in the cryogenic gas of the cryosystem. Isopentane melts at 112–114 K (Fig. 1 ▶) and also supercools and remains liquid below this temperature. Dousing the isopentane with sufficient liquid nitrogen will cause it to freeze (see the video at https://www.youtube.com/watch?v=ZGfJA06Yrwk). The frozen isopentane can be melted by slowly raising the temperature of the gas until it melts. The observed temperature at which an isopentane sample melts gives the temperature of the cryogenic gas with minimal systematic error **at the sample position** and not at some upstream thermocouple within the cryosystem nozzle. Any difference between the temperature readout of the device and the standard melting point of isopentane should be noted, as the device readout must be in error. As discussed later, isopentane can also be used to document the ability of automounters and manual cryotongs to keep samples below the glass-transition temperature when transferring samples from Dewars of liquid nitrogen to the diffractometer.

## 3. Cryoprotectants   

Cryoprotectants are compounds that depress the freezing point of water (and the melting point) and thus help to prevent the formation of ice during the temperature-quenching process of flash-cooling. Fig. 1 ▶ shows some notable temperatures in cryocrystallography to help visualize the temperature ranges involved. Maximizing the cooling rate will be discussed later. Kinetic factors are also important, as discussed in Garman & Schneider (1997 ▶). Typically, crystals of molecules are grown from aqueous solutions of salts, buffers and precipitating agents that do not have suitable flash-cooling properties. That is, if crystals are directly flash-cooled in their mother liquor or growth solutions, then ice can form and damage the crystalline order of the crystals. One solution (pun intended) to overcome this problem is to introduce a cryoprotectant or mixture of cryoprotectants to the crystal before flash-cooling.

Two main characteristics of equal importance are required of a cryoprotectant solution. Firstly, the cryoprotectant solution must vitrify upon flash-cooling without forming any ice. Secondly, the solution must not damage nor degrade the crystal that is being flash-cooled during the time of the procedure. The first criteria is easily tested without an actual crystal in the flash-cooled loop. Studies have shown that a solution that is glass-clear or appears to be vitifried may still exhibit powder diffraction rings from polycrystalline ice, although care should be taken to make sure that a salt in the solution does not also crystallize (Garman & Mitchell, 1996 ▶; Garman & Schneider, 1997 ▶; McFerrin & Snell, 2002 ▶; Chinte *et al.*, 2005 ▶). The same studies have observed that some non-clear flash-cooled cryoprotected solutions do not exhibit powder rings. Therefore, if a cryosystem and a diffractometer are available, solutions without the crystals can be tested beforehand with an X-ray experiment.

The second criterion can be a major stumbling block; nevertheless, a wide variety of cryoprotectants are known to be compatible with protein and other macromolecule crystals (Table 1 ▶), as discussed below. In order to test the second criterion, one requires a crystal and the cryoprotectant solution. The test is simple: put the crystal in the solution or put the solution on the crystal and observe under a microscope whether the crystal cracks, dissolves or is damaged (López-Jaramillo *et al.*, 2002 ▶) as detailed later. Tip: if available, first use a crystal unsuitable for data collection but large enough and of sufficient quality to determine whether physical damage occurs.

Cryoprotectants include small polyols and organics such as glycerol, ethylene glycol, 1,2-propanediol, diethylene glycol, 2-methyl-2,4-pentanediol, dimethyl sulfoxide and other non­volatile alcohols. Volatile alcohols have not been used in the past since they evaporate during the procedure. Indeed, crystals grown in 2-propanol and the like often need to be transferred to a harvesting buffer first. However, recent work by Farley & Juers (2014 ▶) demonstrates how volatile alcohols can be introduced into crystals in the vapor phase and are successful as cryoprotectants. Low-molecular-weight polyethylene glycols (PEGs) such as PEG 400 are suitable cryoprotectants in many instances. Higher molecular-weight PEGs have also been used, although these PEGs have more difficulty than lower molecular-weight PEGs in penetrating into the solvent channels. High concentrations of salts such as lithium formate (Rubinson *et al.*, 2000 ▶) or carboxylic acids such as malonate have also proven to be effective (Holyoak *et al.*, 2003 ▶). Even amino acids such as proline have been used (Pemberton *et al.*, 2012 ▶). One would predict that even some proteins themselves can be cryoprotectants at a sufficient concentration.

Sugars are another suitable choice. Sucrose, trehalose, sorbitol, xylitol and glucose have all been used. A simple technique for creating a cryoprotectant solution with sugars is to make a saturated sugar solution using either the crystal-growth well solution or the mother liquor itself in a small drop next to the crystal-growth drop. This drop volume can be very small: less than a few microlitres. The 100% saturated sugar solution is tested directly and/or diluted to 50 or 75% saturated. A similar technique can be used to make very small amounts of halide solutions for derivatization quick soaks, as shown in the video at https://www.youtube.com/watch?v=45Qc3jOPaKY.

Liquid cryoprotectants such as glycerol and ethylene glycol, when added to an existing solution, will dilute the concentrations of other ingredients such as precipitating agents, salts, additives and buffers. Furthermore, some cryoprotectants, such as glycerol, even increase the solubility of proteins (Vera & Stura, 2014 ▶). A crystal exposed to the newly created solution is unlikely to maintain its integrity. Instead, a new solution with water replaced by the cryoprotectant will need to be made. Newman *et al.* (2008 ▶ and personal communication) have formulated crystallization screening kits with concentrated solutions of conditions that are diluted with water for screening growth conditions and then diluted with additives such as cryoprotectants to screen for cryoconditions. Other cryoprotectants may change the pH or ionic strength and also be unsuitable without taking steps to address these changes. Proteins are generally more soluble in solutions containing glycerol and ethylene glycol, so that one may also need to use higher concentrations of precipitating agent or even higher protein concentrations when making solutions (see below). Sugars should change neither the pH nor the ionic strength of the crystal-growth solution, but at the concentrations typically used will dilute the other solutes, although less than liquid cryoprotectants. Despite the above concerns, a very brief soak and quick flash-cooling may not change the diffraction properties of a crystal appreciably in some instances.

A separate class of cryoprotectants are oils such as perfluoropolyether oil, Paratone-N, olive oil and some others (Hope, 1988 ▶; Riboldi-Tunnicliffe & Hilgenfeld, 1999 ▶; Kwong & Liu, 1999 ▶; Panjikar & Tucker, 2002 ▶). As long as the crystallization buffer does not contain oil-soluble components, these oils are used to coat the surface of a crystal, so that the aqueous layer can be wicked away or removed prior to flash-cooling. If too much water is left on a crystal it can freeze, leading to a damaged crystal and also ice rings in the subsequent diffraction images. If a crystal is dehydrated too much the crystal can be damaged. An oil coating can prevent dehydration when manipulating drops containing crystals (see below).

Oils are nonpenetrating cryoprotectants, as are high-molecular-weight PEGs (Kriminski *et al.*, 2003 ▶). Penetrating cryoprotectants such as glycerol and sugars may enter the solvent channels within the crystal lattice, where together with the protein side chains they may disrupt ice nucleation and freezing (Weik, Kryger *et al.*, 2001 ▶). Therefore, nonpenetrating cryoprotectants such as oils may be less successful with crystals that have a high solvent content and/or large channels, unless a penetrating cryoprotectant is also used.

### 3.1. Decisions, choices, techniques and methods   

**Figure 6:**
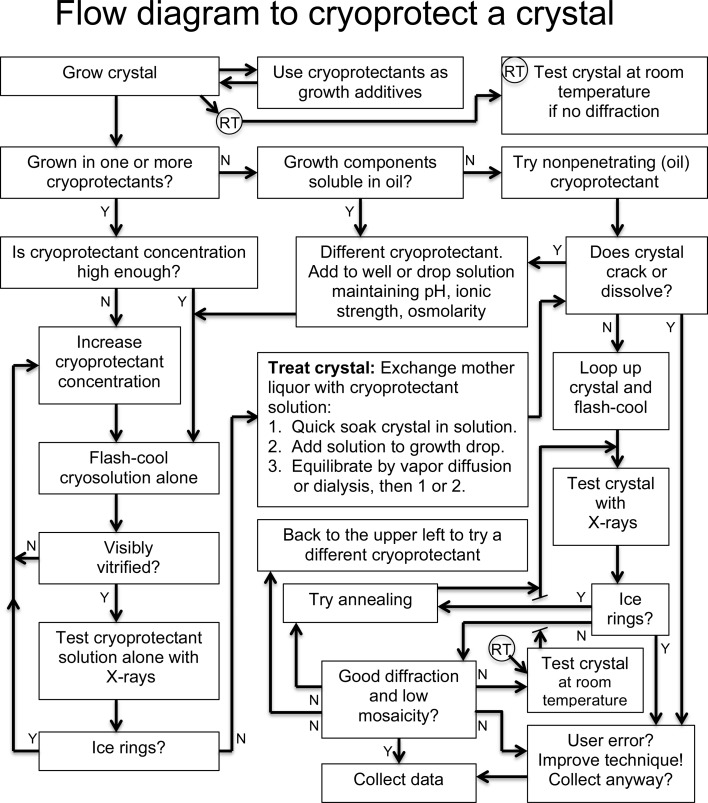
Flow diagram to cryoprotect a crystal. There are two major requirements for a good cryoprotectant solution: (i) it must be vitrified when flash-cooled (left side of the flowchart) and (ii) the crystal must tolerate the cryoprotecting treatment and diffract well when treated with the cryoprotectant solution (right side). Y, yes; N, no. Good technique is also required. Also, a test of crystal diffraction at room temperature is sometimes warranted as noted by the box at the upper right and the circle-enclosed RT symbols.

The above discussion of various cryoprotectants is not meant to be exhaustive. Certainly, not all potential cryoprotectants have been used or even created yet. With so many possible choices, how does one decide which one to start with? And for how much time should a crystal be exposed to the cryoprotecting solution? If a flash-cooled crystal does not diffract well, was the cause the cryoprotectant itself, the technique of introducing it to the crystal or something else? Is a chosen technique reproducible or prone to randomness? These questions, along with some generally successful guidelines for the selection and introduction of cryoprotectants, will now be discussed. A guide for steps to use when cryoprotecting a crystal is shown in Fig. 6 ▶. Similar flow diagrams can be found in other review articles with either less detail (Garman & Owen, 2007 ▶; Rodgers, 2012 ▶) or more detail (Vera & Stura, 2014 ▶).

It cannot be stated enough times that the best results will ultimately be achieved with practice, experience and careful attention to detail.

#### 3.1.1. Grow in cryoprotectant   

Ideally, the process to cryoprotect and flash-cool starts before the crystals are grown. Cryoprotectants can and should be used as additives to optimize crystal growth, even if they are not used at high enough concentrations to be cryoprotective. Cryoprotectants are even crystallization precipitants in their own right. Malonate (Holyoak *et al.*, 2003 ▶; Bujacz *et al.*, 2010 ▶), MPD (Anand *et al.*, 2002 ▶), ethylene glycol, PEG 400, lithium formate (Rubinson *et al.*, 2000 ▶) and others have all been used as primary precipitants while fulfilling a secondary role of disrupting ice formation when the crystal is flash-cooled. Often, these compounds bind to specific sites located on the protein or they create lattice contacts to facilitate crystal growth. If they were not initially present during crystallogenesis, then adding them could induce conformational changes or binding modes that actually disturb the crystal lattice and damage the crystals. Thus, it may be better to have small amounts of cryoprotectant in the crystal-growth conditions, so that increasing their concentration to effective levels has no severe effects. For instance, after an initial screening hit, the hypotheses can be tested that 2, 4 or 8%(*w*/*v*) added sucrose, 5 or 10%(*v*/*v*) added ethylene glycol or 10%(*v*/*v*) MPD either improve crystal growth or at least do not impede crystal growth (with 0% added or the original conditions as a control).

If a cryoprotectant is present in the growth conditions, then a very obvious choice for flash-cooling is to use the same cryoprotectant, perhaps at a higher concentration. Several researchers have tabulated the minimum concentrations of cryoprotectants needed to prevent ice formation and minimize increased crystal mosaicity (Mitchell & Garman, 1994 ▶; Garman & Mitchell, 1996 ▶; McFerrin & Snell, 2002 ▶; Chinte *et al.*, 2005 ▶; Berejnov *et al.*, 2006 ▶; Warkentin *et al.*, 2008 ▶). And, of course, numerous published structure determinations describe the cryoprotectant concentration. Reported cryoprotectant concentrations are concentrated in the ranges 25–40%(*v*/*v*), 25–40%(*w*/*v*) and 40–75% saturated (see Garman & Doublié, 2003 ▶ for a survey of conditions; Berejnov *et al.*, 2006 ▶; Warkentin *et al.*, 2008 ▶). While the concentrations are dependent on the crystal size and the other molecules in the growth medium, a selected concentration is easily tested (see above). As already mentioned, one needs to be aware that higher concentrations of glycerol and ethylene glycol increase the solubility of proteins, so simply adding more of these small polyols without increasing other ingredients or even the free protein concentration may dissolve or damage the crystals (Vera *et al.*, 2011 ▶). In these cases, Vera & Stura (2014 ▶) suggest using ‘cryo-precipitants’ to overcome this effect. However, the reality is that a quick or short soak is often immediately followed by flash-cooling, so that the time for damage to occur is minimized. An increase in protein solubility may be observed from rounding of the edges of a crystal. However, if osmotic shock (for instance from a change in ionic strength) is the problem then the crystal may crack or its surface may become crazed (Garman, 1999 ▶; López-Jaramillo *et al.*, 2001 ▶).

An additional problem with the required cryoprotectant concentrations is that sometimes phase separation occurs in the solutions. For instance, ethylene glycol and salt solutions are not miscible at moderate concentrations. In such cases, two or more cryoprotectants can be used, such as ethylene glycol and sucrose. The use of two or more cryoprotectants at lower concentrations where either alone would be insufficient in a single solution is becoming more common as crystallo­graphers strive to make a universal solution that works in most cases. Vera & Stura (2014 ▶) have created a multicomponent cryoprotectant kit where the use of mixtures of cryoprotectants is stressed. Many commercial crystallization kits now also include more than one cryoprotectant.

#### 3.1.2. Quick soak in a cryoprotectant   

While growing in a cryoprotectant is sometimes a fortuitous option, many crystallization screening recipes do not contain cryoprotectants or crystals simply cannot be grown in the presence of cryoprotectants. The initial crystals may be sufficient to determine the crystal structure if crystal mounting and flash-cooling are successful. Small crystals with minimal mother liquor in contact with them may be flash-cooled directly without prior treatment (Chinte *et al.*, 2005 ▶; Warkentin *et al.*, 2006 ▶). In other cases, the actual elapsed time of soaking of the crystal in a cryoprotectant may be critical for success, whether too short or too long. The crystal does not need to become equilibrated with the cryoprotectant, nor does the cryoprotectant need to penetrate into the crystal for any appreciable amount of time. The aqueous solution on the surface of the crystal generally only needs cryoprotecting or removal, so that a quick soak or swish through the cryoprotecting solution is all that is necessary before the quenching step. The longer that a crystal is in contact with a solution not matched to its growth condition, the higher the chances of damage. Thus, it is prudent to minimize the time spent cryoprotecting before flash-cooling.

The mechanism of a quick soak is typically simply picking up the crystal in a cryoloop or microdevice (attached to a pin + base held by a magnetic wand held in a hand) and placing it in a small amount of the cryoprotectant solution for a moment, then picking it up again in the same manner and plunging the cryoloop (with crystal!) into liquid nitrogen in a flash-cooling step. If dexterity, time and crystal hardiness permits, then any excess surrounding liquid can also be removed or dabbed away.

It may be a daunting task to view a crystal with a microscope and try to capture it with a microloop. The size of the loop plays a role. Sometimes it is easier to use a loop smaller than the crystal like a fork or slotted spoon to manipulate the crystal. Other times a loop that is slightly larger than the crystal works best. The surface tension of the liquid can help as well, with the crystal held in the liquid as the loop is pulled from the drop. One can extract the loop with the plane of the loop perpendicular or tangential to the drop surface or anywhere in between. One is encouraged to practice one’s looping technique with non-precious crystals to see the possibilities. Generally, if the loop plane is perpendicular then less liquid will accompany the crystal (Garman & Owen, 2006 ▶; Pflugrath, 2004 ▶). Slow wafting motions can be better in viscous solutions. Some videos of this procedure can be found at https://www.youtube.com/watch?v=7qg7Z0Gw8Z0 and https://www.youtube.com/watch?v=45Qc3jOPaKY and in Pflugrath (2009 ▶). The reality is that at this moment one is not always thinking about fine details and just wants to get the crystal quickly flash-cooled.

The length of time for the quick soak is quite variable, from 1 to 15 s, since the crystal may escape from the loop holding it and have to be captured again. The procedure places stresses on the crystal, which can crack or bend. The time when the crystal and loop is out of any growth solutions before vitrifying should be minimized since the exposed crystal may either dehydrate or absorb moisture, either of which could have damaging effects (Fig. 1 ▶, dehydration). If the cryoprotectant drop is placed within a millimetre or two of the growth drop then the transfer time is minimized. Longer soaks are un­necessary but sometimes cannot be avoided, although they may not cause any additional harm.

Sometimes even a quick soak does not work and more effort is required for a successful outcome, but one is first encouraged to try the simplest techniques. More involved methods require slow equilibration of the cryoprotecting solution with the crystal-growth solution or matching the osmolarity of the solutions. A few methods of slow equilibration are described next. David & Burley (1991 ▶) placed smaller drops of the new solution near the crystal-containing drop in a vapor-diffusion experiment. Over time, each of the smaller drops was dragged into the drop containing the crystal and thus slowly added the compound to the crystal drop. The vapor-diffusion equilibration helped to reduce any shock caused by unexpected differences in the solutions. Garman (1999 ▶) described adding solutions of increasing glycerol concentrations in 5%(*v*/*v*) steps into a drop with a crystal while removing some volume to keep the total drop volume somewhat constant. The advantages of this method are (i) the crystal is neither touched nor manipulated during the process and (ii) the glycerol concentration increases smoothly owing to mild mixing rather than shockingly stepwise. Others have described dialyzing in the cryoprotectant (Haas & Rossmann, 1970 ▶; Garman, 1999 ▶). As a rule, crystallographers are not so patient unless desperate, so quick soaks seem to be the method of choice. Farley & Juers (2014 ▶) used vapor diffusion to transfer volatile alcohols into loop-mounted crystals.

#### 3.1.3. Nonpenetrating oils   

Oils present their own problems when used as cryoprotectants, but do have many advantages. When a crystallization micro-vapor-diffusion drop is exposed to the atmosphere the drop may start dehydrating, which can affect the crystals within it (Fig. 1 ▶). A barrier or coating of oil will help slow the escape of water (but not volatile components soluble in oil found in the growth conditions) and allow more time for the crystallographer to work with the crystals under a microscope. The goal would be to separate the crystal to be mounted not only from other crystals, but also from any aqueous solution still coating the external surface of the crystal before the oil-coated crystal is flash-cooled. This is easier said than done, especially if stresses on the crystal are to be minimized. A method explicitly shown in Fig. 1 of Warkentin & Thorne (2009 ▶) is to place the ‘wet’ crystal in oil and to stir the oil with the mounting loop without the need to touch the crystal. The motion of the crystal in the moving oil will leave small droplets behind until no more are left on the crystal. Another method is place the wet crystal just outside a drop of oil, then to wick the crystal dry with a dental wick and then to use a loop to quickly drag oil over the now dried crystal. Still another technique is to pick up a wet crystal coated with oil with a loop and dab off the excess oil and water. If the crystal falls off the loop, the crystal is quickly coated with oil again. Often all of these methods are used with the same crystal.

Whatever method is used for cryoprotecting, any excess solution or oil should be removed from the crystal and loop before flash-cooling. Excess solution flash-cooled with the sample increases both the cooling time and the X-ray background during diffraction data collection, but also makes visualizing the sample when trying to align the crystal in the X-ray beam more difficult. Viscous cryoprotectants such as saturated sugar solutions and oils are particularly prone to beading up and remaining with the loop, so that the excesses should be dabbed or wicked away if possible without damaging the crystal. A video of dabbing can be found in the Rigaku webinar on cryocrystallography (Pflugrath, 2009 ▶).

#### 3.1.4. Flash-cooling to cryogenic temperature in a cryogenic liquid   

The actual flash-cooling step should take less than a second of time, but spending some time understanding the final quick step is worthwhile. As previously discussed in the theory section, one wants to flash-cool as quickly as possible so that no ice crystals have a chance to form (but see below). Furthermore, the sample should not inadvertently spend any unnecessary time in the temperature range between the freezing point of ice and the lower bound of the glass-transition phase temperature (Fig. 1 ▶). This can happen if the crystal is held for even a moment in the cold gas above the liquid nitrogen while aiming for a position in a puck or a cryovial rather than plunged as quickly as possible (well under 1 s) into liquid nitrogen, then gently moved around until bubbling stops and finally placed in a puck or cryovial. Even the mere passage of the crystal from the microscope stage to its fate in liquid nitrogen requires passage through several temperature gradients. Warkentin *et al.* (2006 ▶) showed that the cold gas layer above the liquid nitrogen is below freezing and that ice nucleation can occur when the crystal passes through this layer. They suggested that the thickness of the layer could be minimized by blowing dry gas at a temperature above the freezing point of water directly onto the surface of the liquid nitrogen used for quenching. It is also useful to keep a desktop Dewar full of liquid nitrogen so that no cold gas exists inside the Dewar below the rim. All distances and times should be kept as short as possible. As a crystal is plunged into liquid nitrogen it heats up the nitrogen, which boils away. This boiling gas, albeit at cryogenic temperature, certainly lowers the density of nitrogen in direct contact with the crystal and can slow the cooling rate. As the crystal moves quickly downwards it contacts new liquid nitrogen as long as the crystal is kept moving, so generally this gas layer is not an issue (however, see Gakhar & Wiencek, 2005 ▶). In order to avoid this gas layer, other cryogenic liquids with a higher heat capacity than liquid nitrogen may be used, such as tetrafluoromethane or propane. [A simple way to liquefy these gases is described in Pflugrath (2004 ▶): a balloon is filled with gas and its opening is then stretched over a 15 ml plastic centrifuge tube, which is then placed in liquid nitrogen to condense the gas.] In any case, first plunge into liquid nitrogen as quickly as possible and then move the flash-cooled sample into its next resting place, whether in a puck position or a cryovial or cryotongs.

Care should also be exercised so that the orientations of any of the tools, bases or tongs in the liquid nitrogen do not create boil-off or warm gas that comes into contact with the flash-cooled sample. It is not necessary to pre-chill magnetic wands if they are oriented and used with bases away from the crystal loops. However, if a base with a flash-cooled crystal is placed into a cryotong while still warm then the boil-off gas may reach the crystal and create problems. A warm cryotong would be even more disastrous.

#### 3.1.5. Flash-cooling to cryogenic temperature in a cryogenic gas stream   

Another common method of flash-cooling a sample does not involve a Dewar of liquid nitrogen. The cryogenic gas of the cryosystem is used instead. The gas flow is blocked with a plastic or cardboard card (about the width of the cryosystem nozzle is good), the sample is placed on the goniometer head in the mount position and the gas flow is then quickly unblocked, allowing the cryogenic gas to flash-cool the crystal. This works surprisingly well for samples that are adequately cryoprotected. Variations of the theme include turning off the warm dry sheathing gas momentarily. If the gas flow is not blocked first, the crystal may be dehydrated and/or cooled too slowly as it is moved through various temperature regimes into the final mount position. However, gaseous nitrogen is not as dense as liquid nitrogen and the cooling rates are not as fast. There are concerns about the turbulence and mixing of the cold gas with the surrounding air when the cold stream is unblocked. For some crystals, flash-cooling in the stream produces better results than using liquid nitrogen. For others, a specific higher ending temperature was successful (Edayathumangalam & Luger, 2005 ▶).

The entire quick transfer, quick cryoprotectant soaking and flash-cooling procedure is filled with compromises so that it minimizes the stress to the crystal and is as quick as possible. This does not speak to minimizing the stress to the crystallo­grapher. No wonder that sometimes reproducibility suffers or is even unachievable. It is recommended that one practices and perfects one’s technique with less important crystals beforehand. Practice will help increase the ‘yield’ or percentage of successfully flash-cooled, no-ice, low-mosaicity samples available for data collection. For this practice, hen egg-white lysozyme crystals are easy and inexpensive to grow. Recipes for growing lysozyme in a variety of ways in the presence or absence of cryoprotectants are available on the web at http://www.rigaku.com and elsewhere. An assistant can also help with keeping the Dewar filled with liquid nitrogen and covered except during the actual plunging step. A clean dry Dewar of fresh liquid nitrogen should be used after every few crystals to minimize any ice contamination. An extra set or two of dry hand tools are also recommended so that one does not use tools that are wet from condensing moisture. A new dry set is used while the just-used cold and wet set is drying. This helps to keep the liquid nitrogen free of ice.

### 3.2. Transfer to storage or data collection   

The immediate next steps depend on the destination of the flash-cooled crystal. If an automounter is to be used then the crystal will be in the puck designed for use with that automounter. If the diffractometer is off-site, such at as a synchrotron beamline, then the puck will be placed in a dry-shipper Dewar and shipped *via* overnight courier to the diffractometer, where the puck will eventually be placed in the Dewar of liquid nitrogen within reach of the automounter. If the crystal will be manually mounted then sometimes pucks are used, but more often the crystal is placed in a cryovial for temporary storage in liquid nitrogen. Then, when the time comes, the crystal is mounted on the diffractometer. There are two principal methods for this step, both of which try to keep the crystal temperature well below the glass-transition temperature and ice-free. One method requires the gonio­meter head with a magnet to point downwards, so that the cryovial with cryocap can be grasped with cryovial tongs by hand. The cryovial with cryocap can then be moved quickly to the magnet, where the attractive magnetic force will hold the cryocap in place while the cryovial is quickly removed, revealing the crystal to the cryogenic gas flow of the cryosystem. Some devices may be helpful with this technique, such as large removable arcs for goniometer heads (Litt *et al.*, 1998 ▶) or a unique flipping device mounted on the cryonozzle (video at https://www.youtube.com/watch?v=ZGfJA06Yrwk).

The second method requires the use of transfer tongs or cryotongs, as previously introduced above when describing hand tools. Cryotongs were originally described by Parkin & Hope (1998 ▶) and are now produced in commercial versions. Detailed steps for their use are described by Pflugrath (2004 ▶) and at least two videos are available online which show these steps (see above). In summary, the cryotongs are pre-chilled in liquid nitrogen, the tongs are then opened while still in the liquid nitrogen and the cryomount (held by magnetic wand) is placed within the jaws, which are closed around the cryomount encapsulating it. The closed tongs are then moved to the goniometer head magnet, where the base is securely mounted. The closed tongs should prevent any drastic temperature rise inside the tongs and prevent warming of the enclosed crystal to above the glass-transition temperature. Once the crystal base has been properly positioned on the goniometer magnet, the tongs are opened and moved away, leaving the crystal mounted. Care must be taken to do this deliberately without blocking the cryogenic gas flow or exposing the crystal to the outer warm and dry shielding gas. Parkin & Hope (1998 ▶) have shown that one has tens of seconds to complete the transfer. If the crystal is to be removed, the procedure can be reversed. However, since the base is warmer and no longer at liquid-nitrogen temperature and does end up inside the cryotongs, the temperature within the tongs can rise much more quickly during the dismounting than during the mounting steps. Thus, the time allowed for dismounting while keeping the temperature below the glass-transition phase is much, much shorter. The tongs also should be oriented with the handle in line with the gas flow, so that the gas flows between the open halves of the cylinder until the last possible moment of encapsulation. This is also shown in the videos cited previously.

The maximum allowed dismounting time can easily be determined with frozen isopentane (melting point 112–114 K) or 1,5-hexadiene (melting point 132 K) as test samples. Mount a pre-frozen drop of isopentane on the diffractometer and ensure that it remains frozen. The sample of isopentane should then be partially dismounted by (i) grasping with pre-chilled cryotongs, (ii) removing the sample from the goniometer head for a given number of seconds (*e.g.* 3 s) without dunking into liquid nitrogen, (iii) re-mounting the sample on the gonio­meter head and (iv) observing the state of the isopentane. If the isopentane has melted then the time taken was long enough for the sample to exceed the melting point of isopentane, and shorter dismounting times should be used.

Using a similar procedure, frozen isopentane samples can be used to test the dismounting procedure of an automounter. If the isopentane melts then the automounter may be raising the temperature of samples too high during normal dismounts. Such tests have shown that frozen isopentane samples are melted in less time if copper pins and their bases are used than if steel pins with SPINE (see Fig. 4 ▶) bases are used. The conclusion is that more heat is introduced inside the gripper or end effector of an automounter with copper pins than with SPINE pins.

### 3.3. Automounters   

**Figure 7:**
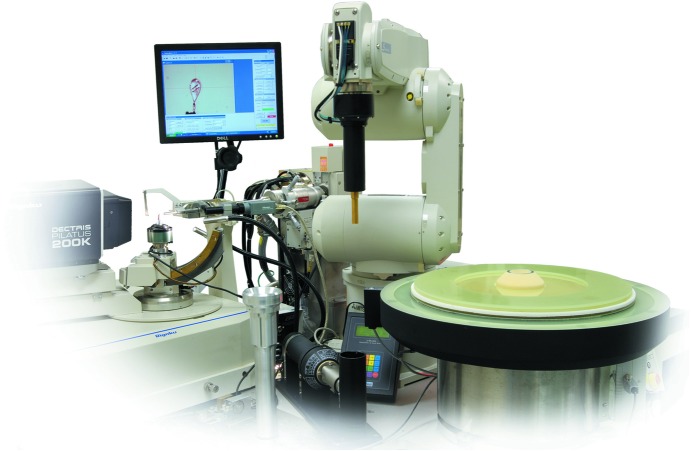
ACTOR from Rigaku. An automated sample-mounting robot with Dewar, robot with end effector, motorized goniometer head and software (photograph courtesy of Rigaku Americas Corp.).

The mechanical procedure of moving a pre-cooled sample from a location in a Dewar containing liquid nitrogen to a fixed location on an X-ray diffractometer lends itself to automation by robots. After pioneering work around the turn of the century at Abbott Laboratories (Muchmore *et al.*, 2000 ▶), Stanford Synchrotron Radiation Laboratory (Cohen *et al.*, 2002 ▶) and the Advanced Light Source/Lawrence Berkeley National Laboratory (Snell *et al.*, 2004 ▶; Cork *et al.*, 2006 ▶), automounters for prepared samples are now used at most single-crystal diffraction synchrotron beamlines and in many home laboratories. An example of an automounter currently used in many laboratories is shown in Fig. 7 ▶. These robots are designed to reproducibly pick up samples held in a pucks under liquid nitrogen with an end effector or gripper similar to the transfer tongs described above and to mount samples in the data-collection position while maintaining the samples ice-free and, most importantly, below the glass-transition temperature. Crystals may be screened for diffraction quality or longer data sets collected before they are dismounted and restored to a location in the Dewar. Typical pucks hold 10–96 samples and Dewars hold 4–6 pucks, so that continuous un­attended or remote operation over hours or days is possible. Indeed, robotic sample mounters have changed the nature of X-ray diffraction data collection, so that automated high-throughput crystallography is no longer fiction. Furthermore, these systems have made remote data collection almost a matter of routine.

Despite advances in automation and high-throughput crystallography, the actual crystal-harvesting, cryoprotecting and flash-cooling steps are still performed manually by dexterous human beings. There are, however, several active research and development programs with the goal of automating even these steps, as reviewed by Deller & Rupp (2014 ▶), but it may be years before their results and devices are widely available. In the meantime, training and practice are recommended to increase the chances of success in flash-cooling.

### 3.4. Annealing, tempering and ice removal   

For those times when a diffraction image from the crystal exhibits ice powder diffraction, a high mosaicity, poorly shaped Bragg spots or other imperfections, the crystal may be annealed or tempered in an attempt to improve the diffraction quality. Annealing consists of allowing the crystal to warm up above the glass-transition temperature range to ambient temperature either by blocking the cryogenic gas flow (Yeh & Hol, 1998 ▶) or by removing the crystal and placing it back into the mother liquor or cryoprotectant solution (Harp *et al.*, 1998 ▶) before a second flash-cooling either in the cryogenic gas flow or in liquid nitrogen. The annealing may be repeated, but be aware that stressing or dehydrating the crystal may damage it further. In these cases, rehydrating in cryoprotectant solution may have some benefit. The success of these methods is likely to stem from the initial fast-cooling procedure being performed suboptimally, such as too slowly or in the cold gas layer found above the liquid nitrogen in a Dewar. If the cryoprotectant used in the initial fast-cooling procedure was inappropriate, then annealing is unlikely to improve the results. Also, one is reminded to observe the crystal during the warming process and to make sure the solution or oil around the crystal actually does liquefy, since the time required to melt will depend on the volume and the exact experimental setup. Perhaps some of the poor results or irreproducibility of annealing arise from a failure to actually warm to ambient temperature. Annealing is successful enough that in many installations it is automated with a blocking card attached to an actuator under computer control. One is also cautioned that sometimes the card interferes with the ability to make sure that the sample melts.

In addition to the relatively simple annealing procedures above, Kriminski *et al.* (2002 ▶) have described a controlled temperature annealing from 150 to 230 or 250 K in 5–10 s. As previously noted, crystal unit cells contract upon cooling to 100 K, with concomitant differences in changes in the density of the solvent and protein regions which induce stress and disorder (Juers & Matthews, 2004 ▶). Kriminski *et al.* (2002 ▶) wrote

Protein lattice stress release and defect migration can occur when water molecules develop significant mobility, which occurs well below water’s freezing point.

This suggests one possible mechanism why annealing works. Juers & Matthews (2004 ▶) discuss net water both flowing into and out of the crystal, which in turn might cause improvements in crystal order and diffraction. Although 250 K is below the freezing point of water, Kriminiski and coworkers noted that crystal-growth conditions with significant concentrations of NaCl can promote low-temperature melting. The Young’s modulus of tetragonal lysozyme has also been reported to decrease as the crystals are warmed, with an abrupt decrease at ∼243 K (Morozov & Gevorkian, 1985 ▶), perhaps caused by the melting of some of the water in the solvent channels of the crystal. Juers & Matthews (2004 ▶) discussed matching of the solvent density and protein density at cryotemperatures. They suggested that annealing would be more successful if the cryoprotectant concentration was too high instead of too low.

If ice is settled onto the surface of the crystal (as seen through a microscope), then the ice may sometimes be washed away by forcefully squirting liquid nitrogen directly onto the sample or by simply rinsing the sample vigorously in liquid nitrogen. Pipettes, pipette tips, plastic tubes, plastic cups and other devices have been used to generate a jet of nitrogen liquid with sufficient force to dislodge the ice from the surface of the mounted sample (videos at https://www.youtube.com/watch?v=Ssgimi3F2JA and in Pflugrath, 2009 ▶). Several automounters can even rinse the sample in liquid nitrogen (Cohen *et al.*, 2002 ▶). Another technique is to brush the ice off with a dental wick or thin fiber, although care must be taken not to thaw the mount by pre-cooling the object first in the downstream cryogenic gas flow. If one anneals the crystals and in the process melts the ice, then the water may dilute the cryoprotectant and other constituents in the previously flash-cooled sample to below useful concentrations (Pflugrath, 2004 ▶). Of course, the best practice is to avoid the ice in the first place, which can be performed by keeping Dewars free of ice from the start.

### 3.5. Recovery, storage and transport   

Removal of samples from the diffractometer has been discussed above. Transfer tongs (Parkin & Hope, 1998 ▶), cryovials holding liquid nitrogen and automounter grippers are the typical devices that are used. Once again, since the pin bases are generally not at cryogenic temperatures when dismounting, the dismount step must be extremely rapid if the crystal is to be kept below the glass-transition temperature. If samples are to be stored for a long time then the samples are stored in racks placed in Dewars of liquid nitrogen. Dry-shipper Dewars, such as the CP100 or CZ100 (TaylorWharton, Minnetonka, Minnesota, USA), are ubiquitous in crystallo­graphy laboratories and single-crystal diffraction synchrotron beamlines. These Dewars are remarkably suitable for use in sample storage and shipping, as evaluated by Owen *et al.* (2004 ▶). Dry-shipper Dewars should be dried to remove adsorbed water from the internal insulating foam after every use for best results. Periodically, the integrity should be checked to ensure that a Dewar still maintains temperature over days. Further advice for maintaining and testing Dewars can be found at the SLAC website: http://smb.slac.stanford.edu/facilities/hardware/cryotools/shipping-dewar-testing.html.

Another practical tip is to always fill a Dewar with liquid nitrogen before removing samples. This is particularly important when extracting a large volume of pucks, since the void left behind will be filled with moist ambient air and thus will introduce ice into the Dewar. Cold dry-shipper Dewars with samples should be filled with liquid nitrogen upon their arrival at the destination to help to minimize internal ice contamination. Another gentle reminder: be cautious and safe when working with liquid nitrogen and Dewars. Always wear personal protective equipment such safety glasses, face shields, gloves and laboratory coats.

### 3.6. Flash-cooling at high pressure   

A method that pressurizes crystals to 200 MPa before flash-cooling has been developed by Gruner and coworkers (Kim *et al.*, 2005 ▶, 2008 ▶). The crystals are first coated with a nonpenetrating oil and then subjected to high pressure before being plunged into liquid nitrogen under the same high pressure. Ice nucleation is suppressed under high pressure, so this technique may be useful as a last resort for some crystals. Among their crystal examples, they used thaumatin and glucose isomerase crystals, which are relatively easy to cryoprotect and flash-cool by conventional methods at normal pressures. However, another advantage is that the crystals flash-cooled under high pressure exhibited lower mosaicity than those cooled under ambient pressure. The oil coating to keep a crystal from dehydrating in the early steps of the procedure may be a disadvantage, since it creates a higher X-ray background, especially for small crystals, and also some mother liquors are soluble in oil. This issue was addressed by using a removable shielding capillary (Kim *et al.*, 2013 ▶).

Another use of high-pressure cryocooling may be for crystals grown in capillaries or small, enclosed devices where they cannot be looped out (Chen *et al.*, 2009 ▶). Clearly, the high-pressure technique has promise when conventional cryoprotecting methods fail. Perhaps with the availability of commercial high-pressure freezing devices at beamlines (Burkhardt *et al.*, 2012 ▶) and the development of automated ways of high-pressure cooling such as the recently published work conducted at the European Synchrotron Radiation Facility (van der Linden *et al.*, 2014 ▶), what is unconventional today will become conventional tomorrow.

### 3.7. Room temperature, capillaries and cryocooling   

Notwithstanding all of the progress made in perfecting the cryocooling of crystals for X-ray data collection, it is still useful to examine diffraction at the crystal-growth temperature to evaluate whether flash-cooling improves or degrades the original crystal diffraction quality. The flow diagram in Fig. 6 ▶ highlights this. As repeatedly noted, macromolecular crystals have a significant solvent fraction, so that any change in the environmental hydration level may stress and damage them. Ever since Bernal & Crowfoot (1934 ▶) first kept pepsin crystals hydrated in thin capillary tubes, the typical room-temperature mounting technique was to insert the crystal into a quartz or glass capillary along with a separated and modest amount of mother liquor or growth reservoir solution (King, 1954 ▶) and then to seal the capillary with wax, glue or even a flame. The nearby liquid would maintain the crystal hydration *via* the vapor phase, much like in the original growth apparatus. Crystals were even grown in capillaries if they were too fragile to mount (Phillips, 1985 ▶). Crystals can even be cryocooled while in capillaries (López-Jaramillo *et al.*, 2001 ▶; Warkentin *et al.*, 2008 ▶). Capillaries have also been slipped over a crystal mounted in a loop (Skrzypczak-Jankun *et al.*, 1996 ▶).

**Figure 8:**
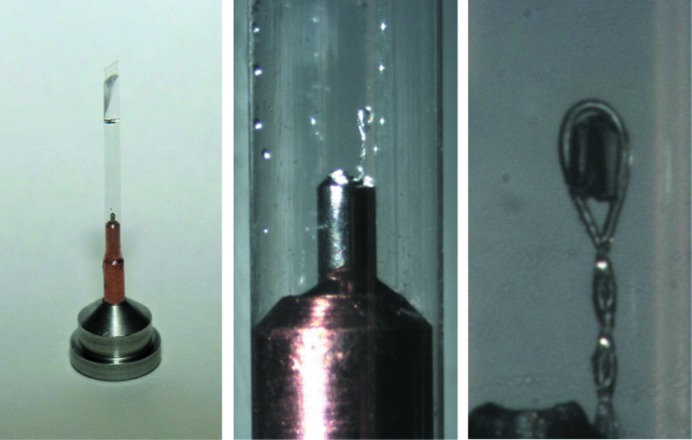
Three views of a crystal mounted at room temperature in a MiTeGen mount with a thin-walled poly(ethylene terephthalate) capillary. Left, full view with a MiTeGen RT base and dual-diameter copper pin. Note the liquid in the top of capillary. Middle, crystal, loop, pin, copper. Right, close-up of the crystal in the loop.

In 2005, the world of capillary-mounted crystals changed when Kalinin *et al.* (2005 ▶) combined cryo-pins on bases with thin-walled poly(ethylene terephthalate) capillaries that would fit snugly over the pins. Fig. 8 ▶ shows three views of a crystal mounted in this device. Together with a microfabricated polyimide mount to replace the nylon loop (Thorne *et al.*, 2003 ▶), these cryo-mounting pins created a simple congruent system. A plastic jig to align the capillary with the pin when mounting made capillary mounting practically foolproof. Thus, one can harvest a crystal from a crystallo­genesis apparatus much in the same way whether for a cryogenic or a room-temperature diffraction experiment. In addition, the capillary is easily removed and the mounted crystal immediately flash-cooled. Therefore, the diffraction quality of a crystal can first be checked at room temperature and then again at cryogenic temperature. In essence, this provides an easy way to check whether poor (or no) diffraction results from the flash-cooling procedure or not. It should be noted, however, that just like with a wet capillary, a crystal on a mount at room temperature can drift or move on the mount during a data-collection experiment, especially if it has too much liquid enveloping it and the orientation with respect to gravity changes. Any unnoticed movement of the crystal will generally reduce the quality of the processing of the diffraction data and even make the data unusable. If the joint between the capillary and mounting is not sealed well, the crystal may also dry out.

### 3.8. Ultralow-temperature helium cryostats   

One question crystallographers have asked is whether even lower temperatures would have a benefit during diffraction data collection, perhaps by reducing the effects of radiation damage. The typical open-flow nitrogen-based cryostat maintains a gas temperature of 90–110 K at the sample. Helium cryostats produce even lower temperatures in the decakelvin range (Schlichting *et al.*, 1994 ▶; Teng *et al.*, 1994 ▶). An open-flow helium cryostat for macromolecular crystallo­graphy without beryllium windows was used with crystals of chicken egg-white lysozyme, sperm whale myoglobin and d-xylose isomerase (Hanson *et al.*, 1999 ▶, 2002 ▶). Lower overall *B* factors for atoms were reported than with higher temperature data collection. A helium cryostat with *T* < 20 K was used by Chinte *et al.* (2007 ▶) to extend the usable lifetime of d-xylose isomerase crystals by a modest 25% during diffraction experiments on the BioCARS beamline 14-BM-C at the Advanced Photon Source. Holton (2009 ▶) noted in studies of metalloproteins that lower temperatures provided by helium cryostats led to lesser radiation damage at their active sites (Yano *et al.*, 2005 ▶; Grabolle *et al.*, 2006 ▶; Corbett *et al.*, 2007 ▶).

Another question that has been asked is do lower temperatures help with the flash-cooling step? Helium does have a much better thermal conductivity than nitrogen, as mentioned by Hanson *et al.* (1999 ▶). Kriminski *et al.* (2003 ▶) discuss the theoretical aspects of flash-cooling in non-nitrogen gases such as helium, ethane and propane. They concluded that any differences in solvent vitrification would be modest with lower temperatures. Larger effects in flash-cooling would arise from the size of the crystal and the amount of excess surrounding liquid, the crystal solvent content, the cryoprotectant concentration and whether plunging into liquid cryogen or a gas stream was used. There has also been some discussion of whether there are other phase transitions in protein crystals when going below the 77 K temperature of liquid nitrogen (Walker *et al.*, 1998 ▶; Parkin *et al.*, 1996 ▶).

Nevertheless, despite continued research in the use of the lower temperatures available with helium devices, it must be noted that worthwhile advantages over the relatively modest temperature of 100 K available with nitrogen are elusive. Also noted as a possible concern by Garman & Owen (2006 ▶) was the cost of using helium, but the expenses described by Chinte *et al.* (2007 ▶) were modest. Perhaps most telling is that of the more than 4700 structural models derived from X-ray diffraction data that were deposited in the Protein Data Bank in 2014, the International Year of Crystallography, only one reported a data-collection temperature between 1 and 75 K and this probably represents a data-entry error. Perhaps more promising is to work at temperatures higher than 100 K, although OH* radicals will be mobile (Owen *et al.*, 2012 ▶). Even room-temperature diffraction data collection on sub­second timescales before too much radiation damage is observed is possible with high-speed detectors at high-brilliance synchrotron beamlines (Warkentin *et al.*, 2012 ▶; Warkentin, Hopkins *et al.*, 2013 ▶; Owen *et al.*, 2014 ▶).

## 4. Discussion and overcoming difficulties   

If the reader has skipped to read this section first in the hope of getting directly to the magic answer to solve their cryocrystallography problems, then they have committed one of the most common errors in the experience of the author: lack of patience. Too often, one has been shown one or two methods for cryoprotecting and flash-cooling which worked well enough to obtain some diffraction data. Then, when difficulties arise one asks colleagues or an internet forum for help. Only after those steps does one consult the literature and finds review articles such as the one being read now and, even then, the entire article may not be read nor the referenced articles consulted for details. Unfortunately, such is the nature of how scientific knowledge is disseminated today.

From the theory presented, it should be apparent that the two main causes of poor results are firstly inappropriate dehydration of or stress on the sample and secondly any amount of time spent in the temperature regime between the freezing point of water and the lower bound of the glass-transition range (see Fig. 1 ▶). These are issues of technique, while a third problem is the choice of the cryoprotecting solution itself. Let us examine each of these in turn.

While dehydration of a crystal is sometimes extremely beneficial (Kiefersauer *et al.*, 2000 ▶), it mostly alters the carefully controlled environment in which the crystal was grown and disrupts the ordered lattice, with concomitant loss of diffraction resolution in an X-ray experiment. The relatively high surface area-to-volume ratio of the crystal and surrounding liquid facilitates both the drying of the sample and water absorption from the environment for hygroscopic samples. The dehydration of crystallization drops is well known in the crystal-growth field, with humidity chambers as standard attachments for crystallization robots. Not surprisingly, studies by Juers and coworkers showed that minimizing dehydration during any of the harvesting and flash-cooling steps led to improved results (Farley *et al.*, 2014 ▶). They used a small humidity-controlled device to blow humid air on their microscope stage when harvesting crystals to keep them from drying out. They also inserted the cryomounts into cryovials with appropriate aqueous liquid to maintain the humidity and O-ring seals to prevent loss of moisture. For flash-cooling in the cryostream, they transported the mounted crystals to the diffractometer in these humidified cryovials. Usually a cold room is quite humid, so sometimes better results simply come from performing the harvesting, looping and flash-cooling in a cold room or even in a walk-in freezer, where the vapor pressure of water is also lower.

The message is clear: work quickly and deliberately in a suitably humid environment. Minimize any distances that bare crystals must move between their growth conditions and their final position in liquid nitrogen. Place the opening of the Dewar of liquid nitrogen as close as possible to the center of the microscope view. Move the mounted crystal as quickly as possible from the microscope into the liquid nitrogen. Practice this step so that one can perform this in less than 0.5 s routinely with confidence. Do not take time to examine the crystal on the loop before flash-cooling. Also, if the crystal is being harvested from a drop then do not allow this drop to dehydrate. Minimize the amount of time that the drop is exposed to ambient air as much as possible. Expose the drop only when ready to harvest and re-enclose it right away or perhaps cover the drop with oil to prevent dehydration. Make sure that the microscope lighting does not also heat the stage. Think like a water molecule sitting in the liquid surrounding the crystal. What keeps you there and what makes you leave?

The quenching or flash-cooling step also needs to minimize the time spent in the temperature regime where ice has a chance to nucleate. Already mentioned earlier is the idea of keeping the sample out of any gas at intermediate temperature. This means keeping the Dewar filled to the brim with liquid nitrogen so that the thickness of the layer of cold gas above the level of the liquid is minimized and by blowing a dry gas source onto the surface of the liquid nitrogen to remove this layer (Pflugrath, 2004 ▶; Warkentin *et al.*, 2006 ▶). Once the crystal has been plunged into liquid nitrogen it is also important to keep it moving to avoid nearby gas bubbles such as when a warm base is also plunged into liquid nitrogen. Keep the crystal positioned lower than the base and pin, especially if placing the cryocap into a cryotong.

All too often, the author has observed half-filled Dewars of liquid nitrogen with ice crystals sloshing around being used in the quenching step. This practically guarantees poor results. For better results, have available some extra Dewars with fresh liquid nitrogen filled to the brim and covered nearby when flash-cooling. Dry and ice-free hand tools are also necessary.

If one is uncertain whether one’s technique is quick enough to keep the sample below the glass-transition temperature, then one can easily test this by using microdrops of isopentane or 1,5-hexadiene as explained previously. If one is assured that one’s skills are well honed, then the cryoprotecting step can be examined more closely. This includes testing the diffraction quality of an ambient temperature-mounted sample in the mother liquor before and after exposure to cryoprotectant.

One is encouraged to separately test, without a crystal, the cryoprotectant solution for its ability to vitrify using the flash-cooling technique chosen. Almost one-third of the flowchart in Fig. 6 ▶ is devoted to this aspect. Obviously, another cryoprotectant can be tried if the originally selected one leads to poor results. While it may make sense to change the class of cryoprotectant first, one should not ignore similar cryoprotectants when screening for better flash-cooling conditions. For instance, the freezing points of high concentrations of glycerol and ethylene glycol are rather different. Furthermore, cryoprotectant molecules can bind to macromolecules in unforeseen ways, so there can be differences between very similar cryoprotectants such as ethylene glycol and glycerol or between sucrose and trehalose or even between canola oil and turbomolecular pump oil.

Mixtures of cryoprotectants should also be considered. Some crystals cannot tolerate the high concentrations needed for a cryoprotectant to be effective, but can tolerate lower concentrations of two or more cryoprotectants which together are quite effective. Kwong & Liu (1999 ▶) used a combination of 5% each of glycerol, glucose, xylitol and ethylene glycol to cryoprotect crystals of carbamoyl phosphate synthetase grown at high salt. They also showed that lower concentrations of penetrating cryoprotectants could be used if a nonpenetrating oil was also used to coat the crystal. These results were echoed by Juers & Matthews (2004 ▶) when they used the penetrating cryoprotectant glucose [60%(*w*/*v*)] together with the non­penetrating oil Parabar 10312 (previously known as Paratone-N) for thermolysin crystals and PEG 400 plus the oil Fomblin YR-1800 for β-galactosidase crystals grown in PEG 8000. Vera & Stura (2014 ▶) also advocate using mixtures of cryoprotectants. They highlighted the need to keep the protein solubility from increasing when using cryoprotectants such as glycerol that are known to increase protein solubility. In order to help to counteract these effects, they suggested adding cryoprotectants that are often used as precipitants to help counteract protein solubility changes, such as MPD, DMSO and polyethylene glycols.

If crystals are too fragile or crack during handling, then gentle cross-linking with glutaraldehyde *via* the vapor phase has been used (Lusty, 1999 ▶; Kwong & Liu, 1999 ▶; Heras & Martin, 2005 ▶).

Perhaps the problem is not with the crystal harvesting or flash-cooling. Even with successful flash-cooling, crystals will still be damaged by radiation. As long as one is quick soaking crystals in a new solution, one may wish to dilute or exchange away buffers, ions and compounds known to increase radiation damage. For instance, when exposed to Cu *K*α radiation from a rotating-anode X-ray generator cacodylate buffer (dimethylarsenate) decomposes and releases a gas. With the relatively high X-ray absorption coefficient of arsenic, this buffer would add to the absorbed dose, so buffer replacement is suggested.

## 5. Conclusion   

Without the advent of cryocrystallography, the impact of high-brilliance X-ray sources such as synchrotron beamlines in macromolecular structure determination would be greatly diminished. Impressively, significant progress has been made in understanding both the causes of radiation damage and how to minimize its effects by cryocooling crystals. In order to make use of this knowledge though, even in the International Year of Crystallography, the watchful safety-glasses-covered eyes and gloved hands of a skilled craftsman still stand between success and failure in obtaining almost every crystallographic model. Except for the step of manually harvesting a crystal from a microdrop of mother liquor and flash-cooling it rapidly to below the glass-transition temperature, almost all of the other steps in the structure-determination process have been successfully automated.

While the entire process is still something to be automated in the future, in the meantime human dexterity and skill are still paramount in order to prevent dehydration and ice formation. In most flash-cooling procedures the human experimenter may have more influence on the variability of results than the choice of cryoprotectant or the method of flash-cooling, no matter whether the sample is plunged into cryogen or quenched in a cryogenic gas stream. Careful attention to detail, dedicated practice and deliberate speed are all well rewarded until even this step becomes automated. A little knowledge of the physics of the method does not hurt as well.

## Figures and Tables

**Figure 3:**
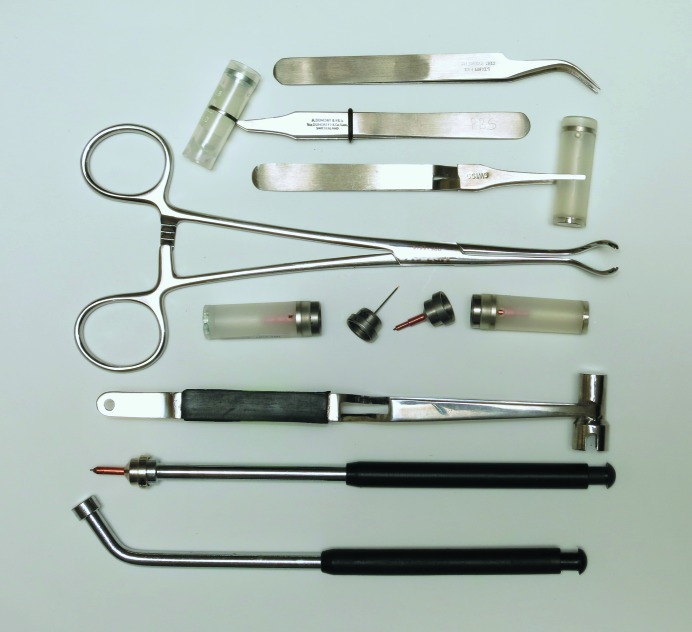
Hand tools for cryocrystallography. From the top: forceps for grasping mounting pins; cryovial forceps with O-ring to maintain closure; self-closing forceps to hold vials and mounts without finger pressure; cryovial locking tongs; cryovials and cryobases with pins and loops; cryotongs; straight magnetic wand; curved magnetic wand. Items are from Hampton Research, MiTeGen and Molecular Dimensions

**Table 1:** An inexhaustive list of cryoprotectants (or ‘What’s on your shelf?’) Cryoprotectants in bold are very commonly used.

Class	Cryoprotectant	Concentration	Comments	Reference†
Small polyols	**Glycerol**, **ethylene glycol**, propylene glycol, 1,2-propanediol, diethylene glycol, **2-methyl-2,4-pentanediol**, 2,3-butanediol, 1,6-hexanediol	2550%(*v*/*v*)	Replace water, do NOT dilute out other buffer components	Garman Mitchell (1996 ▶), Berejnov *et al.* (2006 ▶), Vera *et al.* (2011 ▶), Vera Stura (2014 ▶), Anand *et al.* (2002 ▶)
Alcohols	Methanol, ethanol, 2-propanol		Use *via* the vapor phase or avoid since too volatile	Farley Juers (2014 ▶)
Sugars	**Sucrose**, trehalose, glucose, sorbitol, xylitol, erythritol, inositol	5075% saturated	Use in mother liquor or reservoir solution	Haas Rossmann (1970 ▶)
Organic salts	Malonate, formate, acetate	50100% saturated	Adjust pH of stock solutions to neutral	Holyoak *et al.* (2003 ▶), Rubinson *et al.* (2000 ▶)
Inorganic salts	Lithium nitrate, lithium sulfate, lithium chloride, sodium nitrate	5090% saturated	Lithium is incompatible with phosphate	Rubinson *et al.* (2000 ▶)
Low-molecular-weight PEGs	PEG 200, **PEG 400**, PEG 600 *etc.*	2550%(*v*/*v*)		
High-molecular-weight PEGs	PEG 3350, PEG 6000, PEG 8000 *etc.*	2550%(*w*/*v*)	Nonpenetrating	
Amino acids	L-Proline	23*M*		Pemberton *et al.* (2012 ▶)
Osmolytes	Trimethylamine *N*-oxide, sarcosine, betaine	4*M*		Mueller-Dieckmann *et al.* (2011 ▶), Marshall *et al.* (2012 ▶)
Others	Dimethyl sulfoxide	30%(*v*/*v*)	Helps with solubilizing small ligands	
Oils	Parabar 10312, **perfluoropolyether oil**, turbomolecular pump oil, paraffin oil, canola oil, olive oil	Very thin coating of the crystal, so dab away excess oil to reduce X-ray scatter	Parabar 10312 is also known as Paratone-N and is more viscous than the other oils	Riboldi-Tunnicliffe Hilgenfeld (1999 ▶), Panjikar Tucker (2002 ▶)
Mixtures	Parabar 10312 + perfluoropolyether oil, polyols + sugars + PEG polyols, then oil		Mixtures of cryoprotectants are limited only by one’s imagination	Juers Matthews (2004 ▶), Vera Stura (2014 ▶)

† References are meant to be suggestive and not exhaustive.
